# Erratum: Wise et al., “Prolonged Activity Deprivation Causes Pre- and Postsynaptic Compensatory Plasticity at Neocortical Excitatory Synapses”

**DOI:** 10.1523/ENEURO.0432-24.2024

**Published:** 2024-10-11

In the article “Prolonged Activity Deprivation Causes Pre- and Postsynaptic Compensatory Plasticity at Neocortical Excitatory Synapses,” by Derek L. Wise, Yasmin Escobedo-Lozoya, Vera Valakh, Emma Y. Gao, Aishwarya Bhonsle, Qian L. Lei, Xinyu Cheng, Samuel B. Greene, Stephen D. Van Hooser, and Sacha B. Nelson, which was published online on May 22, 2024, Berith Isaac was omitted from the author list. Dr. Isaac should appear fourth in the author line, between Vera Valakh and Emma Y. Gao, and should be credited with designing research, performing the research, analyzing data, and writing the paper in the contributions statement. The corrected author and affiliation line and author contributions appear below, and the online version has been corrected.

Derek L. Wise, Yasmin Escobedo-Lozoya, Vera Valakh, Berith Isaac, Emma Y. Gao, Aishwarya Bhonsle, Qian L. Lei, Xinyu Cheng, Samuel B. Greene, Stephen D. Van Hooser, and Sacha B. Nelson

Department of Biology, Brandeis University, Waltham, Massachusetts 02454 9110

Author contributions: D.L.W., Y.E.-L., V.V., B.I., S.D.V.H., and S.B.N. designed research; D.L.W., Y.E.-L., V.V., B.I., E.Y.G., Q.L.L., X.C., and S.B.G. performed research; D.L.W., Y.E.-L., V.V., B.I., A.B., X.C., S.B.G., S.D.V.H., and S.B.N. analyzed data; D.L.W., Y.E.-L., V.V., B.I., S.D.V.H., and S.B.N. wrote the paper.

