# Supplementary figures and images for: Prolonged Activity Deprivation Causes Pre- and Postsynaptic Compensatory Plasticity at Neocortical Excitatory Synapses
Source: eNeuro. 2024 Jun 6;11(6):ENEURO.0366-23.2024. doi: 10.1523/ENEURO.0366-23.2024 (PMC11163391; doi:10.1523/ENEURO.0366-23.2024)

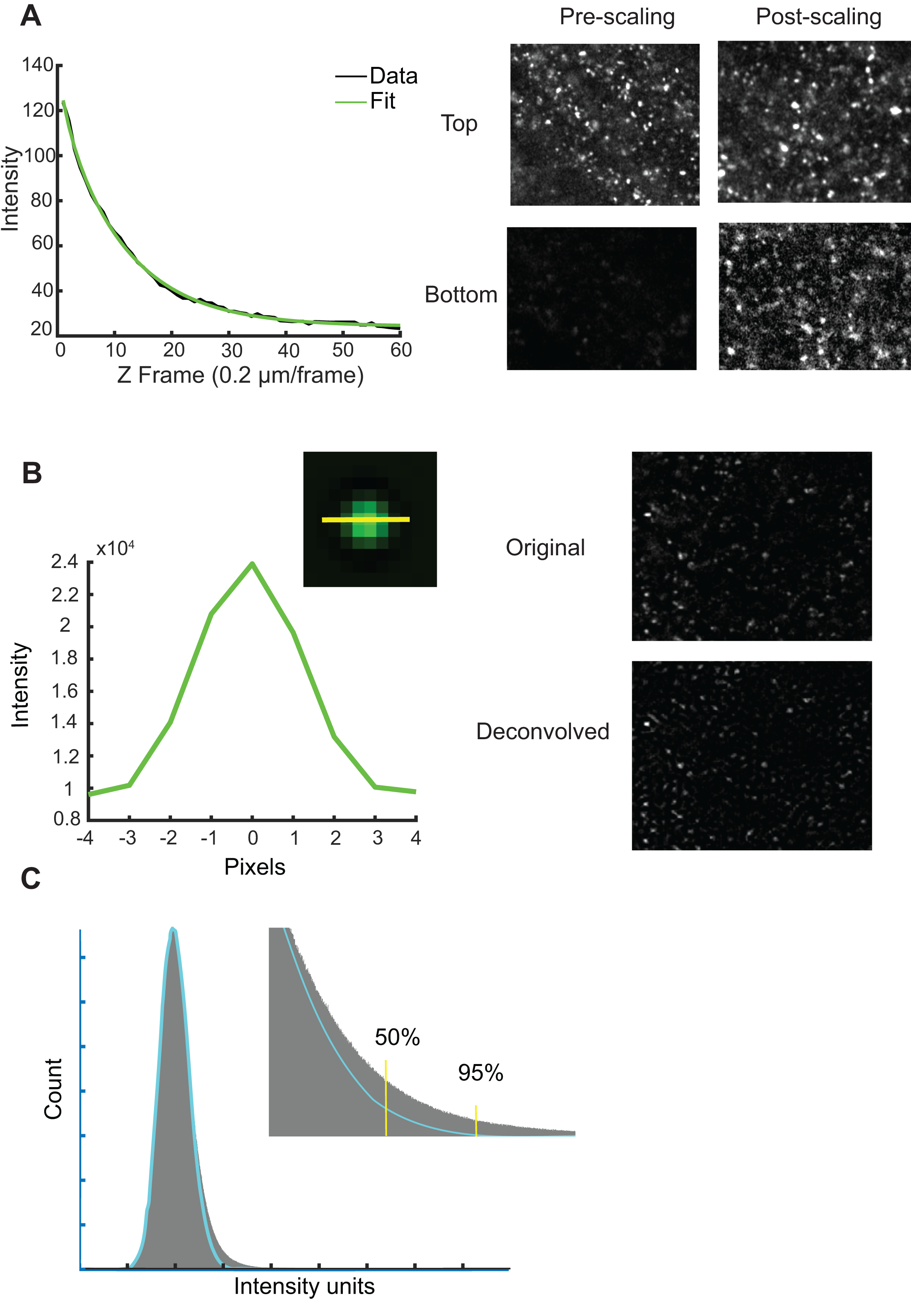

Supplement: Figure 3-1 — Download Figure 3-1, TIF file. [file eneuro-11-ENEURO.0366-23.2024-s002.tif]
